# Development and Validation of the Family and Community Nursing Advanced Practice Scale

**DOI:** 10.1111/jocn.17665

**Published:** 2025-01-20

**Authors:** John Unsworth, Crystal Oldman, Joanne Atkinson, Dania Comparcini, Valentina Simonetti, Giancarlo Cicolini, Kristina Mikkonen, Marco Tomietto

**Affiliations:** ^1^ Department of Nursing, Midwifery and Health, Faculty of Health and Life Sciences Northumbria University Newcastle upon Tyne UK; ^2^ Section of Nursing, Department of Precision and Regenerative Medicine and Ionian Area University of Bari "Aldo Moro" Bari Italy; ^3^ The Queen's Nursing Institute London UK; ^4^ Department of Social Work, Education and Community Wellbeing, Faculty of Health and Life Sciences Northumbria University Newcastle upon Tyne UK; ^5^ Section of Nursing, Interdisciplinary Department of Medicine “Aldo Moro” University of Bari Bari Italy; ^6^ Department of Innovative Technologies in Medicine & Dentistry “G.d'Annunzio” University of Chieti Pescara Italy; ^7^ Research Unit of Health Science and Technology University of Oulu Oulu Finland; ^8^ Medical Research Center Oulu Oulu University Hospital, University of Oulu Oulu Finland

**Keywords:** advanced practice nursing, community, family nursing, roles, scope of practice

## Abstract

**Aim:**

To develop and test a Family and Community Nursing—Advanced Practice Scale.

**Design:**

A cross‐sectional and methodological scale validation design, following classical test theory.

**Methods:**

Three phases, the first of which involved scale development, including item generation. Phase two assessed the content validity index. The third phase involved a cross‐sectional survey to establish construct validity, content validity, internal consistency reliability, and exploratory factor analysis.

**Results:**

The Family and Community Nursing Advanced Practice Scale has good construct validity, with the final scale consisting of 5 domains and 27 items. This was confirmed by both the exploratory and confirmatory factor analysis. The Cronbach's Alpha is very good, suggesting that the scale is reliable. When comparing family practice advanced practice nurses with those working in the community, the results show that scores are similar except for clinical reasoning and health promotion, which consistently showed statistically significant higher scores among the family practice nurses. While community nurses scored higher on items in the leading practice domain reflecting their role in a wider team of nurses.

**Conclusion:**

This study developed and psychometrically tested the Family and Community Nursing—Advanced Practice Scale. The scale has good reliability, and analysis of the construct validity reveals five domains of advanced practice among this practitioner group.

**Implications for the Profession:**

The study suggests that advanced practice nurses working in community roles perform similar activities to those working in family practice in the United Kingdom. However, activity related to research was less evident.

**Impact:**

The study examined the scope of the advanced practice nurse role in family and community nursing. The study illustrated practice across five domains: clinical care, leading practice, clinical reasoning, health promotion, and ethics. The family practice and wider community roles were largely homogenous, with only two items showing a statistically significant difference in scores.

**Reporting Method:**

STROBE guidelines for cross‐sectional studies.

**Patient or Public Contribution:**

No patient or public contribution.

**Summary**

- What does this paper contribute to the wider global clinical community?
  ○ This study is one of the first to examine the frequency of engagement in the various domains of advanced practice, showing homogeneity between advanced practice nurses working in different community settings.
  ○ Advanced practice nurses working in family practice and those working in the wider community have similar roles and perform a similar range of functions around patient management and chronic disease.
  ○ The research domain of advanced practice nurses is less well developed among those practicing in family practice and the wider community than the domains around education and leadership.

## 1 Introduction

Advanced practice nursing originated in the 1960s in the United States and has grown exponentially across the world with the development of roles across every major continent (Wheeler et al. 2022). The growth of roles is a response to increasing patient demand and unmet needs, particularly concerning Universal Health Coverage (UHC) and promoting timely access to care (Lopes‐Júnior 2021). The growth is also driven by population changes, demographic and epidemiological trends resulting in an increasingly ageing population and a rise in the number of people living with multiple long‐term conditions (Bales et al. 2023). Advanced practice nursing roles are commonly found in family practice (called general practice in the United Kingdom) and community services. Such roles are central to the provision of universal health coverage by providing care to either the general population or specific groups within the population. While some roles are designed to substitute the role traditionally performed by a family doctor (Strachan et al. 2022) other roles provide more specialised services or services to marginalised communities such as people who are experiencing homelessness (Ungpakorn, Sehmbi, and MacLaine 2021).

The International Council of Nurses (2020) defined an advanced practice nurse as either a specialist or generalist who, through master's education, has acquired expert knowledge and the competencies required to make complex decisions and perform as a nurse at an advanced level. The International Council of Nurses (2020) describes how advanced practice nurses practice at a level beyond the scope of practice of a generalist or specialist nurse with the capability to manage full episodes of care, greater autonomy, advanced judgement, decision‐making, and diagnostic reasoning skills with the authority to diagnose, prescribe medicines, order diagnostic tests, refer, and admit/discharge patients. From an initial role in primary care, advanced practice nurses have developed across a wide range of specialties, including in emergency, critical, ambulatory, and long‐term care (Kleinpell 2009). As advanced practice nursing has evolved globally, a wide variety of different nomenclatures are used for advanced practice roles (De Raeve et al. 2024), and it is often better to think of advanced practice as a level of practice rather than a distinct role.

Globally there is little heterogeneity regarding title use, regulation, and the scope of practice of advanced practice nurses (Beckmann et al. 2023). Countries such as the Republic of Ireland have regulations for Advanced Nurse Practitioners, while the United States has produced a consensus model around the Advanced Practice Registered Nurse (APRN) title. The United Kingdom currently has no system of regulation, although various professional bodies have offered credentialing (Royal College of Nursing 2020). Even where regulation exists, it is often applied to some groups and not others, such as in Ireland, where specialist nurses are not regarded as advanced practice nurses. In addition, the APRN role is not universal across all US states and territories with varying levels of practice authority (National Council of State Boards of Nursing 2024).

In the United Kingdom a wide variety of titles are in use, including Advanced Nurse Practitioner (ANP), Advanced Clinical Practitioner (ACP), and Nurse Practitioner (Fothergill et al. 2022). An Advanced Nurse Practitioner is defined as ‘an experienced and autonomous registered nurse who has developed and extended their practice and skills beyond their previous professional boundaries. The ANP can use their expert knowledge and complex decision‐making skills in unpredictable situations. This includes managing patients with undiagnosed health care problems and is shaped by their context of practice’ (Royal College of General Practitioners 2015, p. 6). An Advanced Clinical Practitioner has a similar role, but the title represents a multi‐professional role involving allied health professionals, pharmacists, and paramedics as well as nurses (Timmons et al. 2023).

The lack of heterogeneity among advanced practice nursing roles has led to researchers seeking to delineate roles through a process that allows for the measurement of nursing activity, the scope of practice, as well as identifying which nurses align with the advanced practice nursing criteria (Gardner et al. 2013).

## 2 Background

Globally, several studies have sought to evaluate the scope of practice of advanced practice nurses or to delineate advanced practice nursing from other levels of practice (Gardner et al. 2016; Jokiniemi et al. 2022; Carryer et al. 2018). In addition, some studies have sought to examine the differences in practice between nurse practitioners and clinical nurse specialists (Gardner et al. 2016; Jokiniemi et al. 2021; Carryer et al. 2018). A variety of approaches, have been used including Delphi surveys, cross‐sectional survey approaches and the development and application of role delineation scales.

One of the most commonly used scales is the Advanced Practice Role Delineation Tool (APRD Tool) (Gardner et al. 2016). The APRD Tool is based on the Strong Model of Advanced Practice, which was originally developed as part of the emerging acute care nurse practitioner role (Ackerman et al. 1996). The tool consists of 41 activity items across five domains of practice: direct care, support of systems, education, research and publication, and professional leadership. Respondents indicated the extent to which they undertake each of the 41 items using a 5‐point Likert scale from 4 = a very great extent; 3 = great extent; 2 = some extent; 1 = little extent; to 0 = not at all. While initial small‐scale pilot work was conducted by Mick and Ackerman (2000), the first main study was used to differentiate advanced practice nurses with a variety of different titles, including clinical nurse consultant, clinical nurse specialist, and nurse practitioner, across all states and territories in Australia. Gardner et al. (2016) found that nurses working at an advanced level had high mean scores across all five domains of practice. The researchers also found that the mean scores for advanced practice nurses were significantly higher for nurse practitioners in the direct care domain and that scores in the other four domains were higher for advanced practice nurses than registered nurses, educators, and managers.

Jokiniemi et al. (2022) used the APRD Tool to study advanced practice nurses in Hong Kong, comparing advanced practice nurses and nurse consultants. They found that nurse consultants had higher mean scores across all five domains with significantly higher mean scores for support for systems, education, research and publication, and leadership.

In New Zealand, Carryer et al. (2018) used the APRD Tool to examine the differences between nurse practitioners, clinical nurse specialists, and registered nurses. They found that nurse practitioners and clinical nurse specialists had similar domain scores, with nurse practitioners has slightly stronger mean scores for direct care and professional leadership than clinical nurse specialists.

In addition, the APRD Tool was used as the basis for a Finnish modified version of the Strong Model of Advanced Practice (MoSMAP), which consisted of 45‐items across five domains (Jokiniemi et al. 2021). The MoSMAP tool was subsequently used to collect data from registered nurses, registered midwives, clinical nurse specialists, and nurse practitioners employed in five universities and one central hospital in Finland. The results showed that registered midwives had the highest score for direct care, followed by registered nurses. Clinical nurse specialists had higher scores in support for systems, research and publication, and leadership. This study highlights how roles differ in different health systems and contexts. Jokiniemi et al. (2022) also conducted a confirmatory factor analysis on the APRD Tool and the MoSMAP and found that the 45‐item MoSMAP tool has better psychometric properties and construct validity than the original 40‐item APRD Tool.

The Advanced Practice Nursing Competency Assessment Instrument (APNCAI) was constructed using a Delphi survey approach (Sastre‐Fullana et al. 2017) and is made up of eight factors: research and evidence‐based practice, clinical and professional leadership, interprofessional relationship and mentoring, professional autonomy, quality management, care management, professional teaching and education, and health promotion. The instrument consists of 54 items and while it has been psychometrically evaluated, it has not been used to compare advanced practice nurses in different settings or roles (Sastre‐Fullana et al. 2017; Dias et al. 2022). The APNCAI is more widely used to identify and assess the competencies of advanced practice nurses rather than to evaluate their scope of practice and specific roles in the healthcare setting. Similarly, the Nurse Practitioners' Roles and Competencies scale (Lin et al. 2015), which consists of 51 items across six domains, including professionalism, direct care, clinical research, practical guidance, medical assistance, and leadership and reform, was developed to consider the role of nurse practitioners in Taiwan. Lin et al. (2015) describe how the scale can be used to assess the competencies of nurse practitioners in Taiwan and as a reference for other Asian countries seeking to develop the role.

Other tools have been developed to explore specific aspects of the advanced practice nurses' role. Sebach (2022) developed and psychometrically tested a tool examining the nurse practitioners' clinical educator competence. The Academic Clinical Nurse Educator Skills Acquisition Tool (ACNESAT) is a 40‐item tool consisting of two factors: facilitating clinical learning through teaching, assessment, and evaluation and promoting nursing enculturation. The tool was a useful measure of competence for nurse practitioners in the educational dimensions of their role.

A range of tools and scales have been developed to assess the work of advanced practice nurses. Many of the tools have been developed to assist in role delineation (Gardner et al. 2016; Jokiniemi et al. 2022; Carryer et al. 2018), while others are more generic, exploring the scope of practice of advanced practice nurse roles (Chang et al. 2010; Sastre‐Fullana et al. 2017; Lin et al. 2015). There has been a dearth of work exploring the scope of practice and role of advanced practice nurses in different clinical settings. A study by Jafari Pour et al. (2024) identified that advanced practice nurses in critical care spend the majority of their time in direct care with less emphasis on education, leadership, and support for systems. To some extent this is understandable given the complexity of clinical patient management in critical care. There is no published work on the scope of practice of the advanced practice nurse in family practice or the wider community, even though the role extends into population‐ and community‐based health as well as direct care for individuals (Royal College of General Practitioners 2015). To address this gap, this study aims to explore the role and scope of practice of advanced practice nurses working in family (general) practice as well as those in wider community roles.

## 3 The Study

This study employed a cross‐sectional and methodological scale validation design, following the Classical Test Theory (CTT) (DeVellis and Thorpe 2021).

The study was conducted to develop and test a new Family and Community Nursing—Advanced Practice Scale (FCN‐APS). The research consisted of three phases, the first of which involved scale development, including item generation. Phase two then assessed the Content Validity Index (CVI) with an expert panel. The third phase involved a cross‐sectional survey to establish construct validity, content validity, internal consistency reliability, and exploratory factor analysis. The aim of the study was to develop and psychometrically test a scale that could measure the scope of practice and frequency of activities among a broad range of advanced practice nurses working in family practice and wider community roles. At the same time, the research team wanted to explore whether the profile of activities differed between those advanced practice nurses who worked in family (general) practice and those who worked in wider community roles.

## 4 Methods

### 4.1 Aims and Questions

The aim of the study was to develop and psychometrically test a scale that could measure the scope of practice and frequency of activities among advanced practice nurses working in family practice and community roles.

The specific research questions were:

- What is the scope of the advanced practice nurse role working in family practice or a community setting in the United Kingdom?
- How frequently do advanced practice nurses report engaging in each of the domains of their role?
- Is there any difference between advanced practice nurses working in the wider community when compared to those who work in family (general) practice?

### 4.2 Scale Development

#### 4.2.1 Phase I: Item Generation

Item generation used both deductive and inductive methods. Existing models of advanced nursing practice, literature about the role of the family and community nurse, and existing competency frameworks were used to generate an initial range of 36 items. Once generated, these items were then mapped to the Hamric model (Hamric, Spross, and Hanson 1996), the four pillars of advanced practice (Health Education England 2017), the Nursing and Midwifery Board for Ireland domains (Nursing and Midwifery Board of Ireland 2017), and the ESCO competencies (European Commission 2017). An initial panel of 6 experts then reviewed the items relevance and readability, suggesting items be reworded for clarity to avoid ambiguity and colloquialisms and to split double‐barreled items. Revised items were then checked to remove any duplicates, and the mapping to the models and frameworks was revised. The final total item list was 41 items.

#### 4.2.2 Phase II: Content Validity

Once the items had been agreed upon by the research team, a panel of 10 clinical advanced practice nurses from family (general) practice and community services was asked to evaluate the content and construct validity by scoring each item for relevance using a 4‐point Likert scale from not relevant to very relevant and also for clarity using a 4‐point Likert scale from not clear to very clear. Each item was considered relevant or clear if the experts rated it as 3 or 4 on the Likert scale (relevant/very relevant and clear/very clear). Minor corrections were made to the statements following expert feedback; these related to adding family and community to some of the statements. This phase established the content validity of the scale by calculating the Content Validity Index at the scale level (S‐CVI). The S‐CVI was calculated by considering the average of the items' Content Validity Index (I‐CVI). The cut‐off value for the S‐CVI is 0.83 (Polit and Beck 2006).

#### 4.2.3 Phase III: Psychometric Testing

In phase 3, the scale was administered to a sample of FCN‐Aps, and descriptive statistics, internal consistency, EFA, and CFA were calculated to assess the psychometric characteristics of the scale and achieve adequate reliability and construct validity. The scale, consisting of 41 items, was constructed alongside a 5‐point Likert response scale for each item from (Britain Thinks 2023) ‘very great extent’; (Beckmann et al. 2023) ‘great extent’; (Bales et al. 2023) ‘some extent’; (Mick and Ackerman 2000) ‘little extent’; and (Ackerman et al. 1996) ‘not at all’.

### 4.3 Study Setting and Sampling

The target population was constituted by those registered nurses with an advanced practice qualification within the United Kingdom working as either an Advanced Nurse Practitioner, Advanced Clinical Practitioner or Nurse Practitioner in family or community practice. There is no register of individuals with this qualification and therefore the total population size is difficult to estimate. Participants were eligible to take part in the survey if they held an advanced practice qualification and at least 1 year of clinical practice as an advanced practice nurse. The participants were included if they were in clinical practice at the time of data collection. The sample size calculation considered a participant‐to‐item ratio ranging from 5:1 to 20:1 (Kline 2023). In detail, to properly perform the Exploratory Factor Analysis (EFA), it is adequate to consider at least 100 participants and a 5:1 ratio. For a Confirmatory Factor Analysis (CFA) it is recommended to recruit at least 10 participants per item (Kline 2023). Hence, given the number of 41 items in the initial development of the scale and the need to perform both an EFA and a CFA in phase 3, the required sample size was of 410 participants.

### 4.4 Data Collection

A convenience sampling criterion was applied to collect data. An online survey was developed in the Joint Information Systems Committee data technology provider for UK Higher Education (JISC) survey platform, and periodic reminders via professional channels were planned to maximise participation and the participants' engagement with the project. This included emails to network members and promotion via social media and newsletters. The survey took place from May to July 2024.

### 4.5 Data Analysis

The data were analysed using IBM SPSS v28.0 for descriptive statistics and EFA and Stata v13 for CFA.

Preliminary analyses were performed to meet the assumptions to properly calculate the multivariate statistics according to the CTT. In detail, the Mahalanobis distances and the p‐values in the chi‐square distribution were calculated to identify multivariate outliers. If the multivariate normality is not verified, identifying and removing multivariate outliers is essential to meet the assumptions for calculating multivariate statistics (Kline 2023). Mardia's kurtosis coefficient was calculated to check multivariate normality. In detail, multivariate normality is verified if Mardia's kurtosis coefficient is lower than the critical value v*(v + 2) (where “v” equals the number of items) (Mikkonen, Tomietto, and Watson 2022). No missing values were observed.

Psychometric testing was performed by calculating Cronbach's alpha, McDonald's omega, and their values if an item was deleted from the scale, and item‐to‐total correlations. Regarding Cronbach's alpha (*α*), scores > 0.90 are considered excellent, scores between 0.70 and 0.90 are considered good, scores between 0.60 and 0.70 are acceptable, and values below 0.60 are non‐acceptable (DeVellis and Thorpe 2021). McDonald's omega (ω) is recommended alongside Cronbach's alpha as it considers items' loadings within the computation of reliability, and it is a more robust score for reliability. McDonald's omega values > 0.60 are considered “acceptable”, even if some authors recommend values > 0.80 (Mikkonen, Tomietto, and Watson 2022). The variation of Cronbach's alpha and McDonald's omega if each item was deleted was calculated. If the score increases over 0.10, then the item should be deleted, as it does not contribute to the scale's reliability. Item‐to‐total correlation was also calculated; if lower than 0.30, then the item should be considered for deletion (DeVellis and Thorpe 2021).

Construct validity was tested by calculating an EFA with a principal axis factoring approach and direct oblimin rotation, as this scale assesses behavioural and psychosocial components and factors correlated > 0.2 with one another (Mikkonen, Tomietto, and Watson 2022). The eigenvalues' cut‐off was initially set at 1. The Kaiser‐Meyer‐Olkin (KMO) test and the Bartlett's test of sphericity were used to verify the sample's adequacy. Specifically, KMO values between 0.8 and 1.0 and a statistically significant Bartlett's test indicate the sample is adequate for an EFA (DeVellis and Thorpe 2021). Items were considered for deletion if the item's loading was < 0.30 or the cross‐loading difference was < 0.20. The cross‐loading difference was calculated by considering the absolute value of the loadings and the difference between the first and second highest loading of each item (DeVellis and Thorpe 2021).

To test the construct validity of the final factorial solution, a CFA and its fit indices were calculated. The fit indices are considered acceptable for RMSEA (root mean square error of approximation) and SRMR (standardised root mean residual) < 0.08, and CFI (comparative fit index) and TLI (Tucker‐Lewis index) > 0.90 (Kline 2023).

Categorical data were expressed as frequencies and percentages. Mean, standard deviation (SD), minimum, maximum, skewness, and kurtosis were calculated for continuous variables and the scale's items.

### 4.6 Ethical Considerations

The procedures for data collection and analysis were designed to guarantee data confidentiality and comply with both national and European regulations, including the GDPR (2018) and the UK Data Protection Act (2018). Electronic data were securely stored in a protected folder, with access limited to the principal investigator and the research team. Participants received a disclaimer on the initial survey page detailing the study and data handling procedures. Submission of the survey constituted their consent to participate in the study. Ethical approval was obtained from the university (ref: 7020, 19/04/2024).

## 5 Results

### 5.1 Phase 1: Item Generation

A total of 41 items were generated and then mapped back to the original models with 14 items in the direct clinical care and decision‐making domain, 3 in communication, 3 in evidence‐based practice 3 in ethical decision‐making, 6 in guidance and prevention, 3 in collaboration, 3 in educating others, and 6 in team leadership and management (Table 1). Each item was considered by the research team and revised to ensure that it was linked to a single domain and was easily understood. Table 1 reports the items generated in this phase and their link to Hamrich's model (Hamric, Spross, and Hanson 1996), the Nursing and Midwifery Board for Ireland model (Nursing and Midwifery Board of Ireland 2017), and the ESCO model (European Commission 2017). The research team included three academics who were experts in community advanced practice and one policy expert. The remaining academics had expertise in psychometric measurement and tool design.

**TABLE 1:** Items' generation and link to the international models of advanced competencies.

FCN‐APS components	Hamric's integrative model	NMBI	ESCO – European skills and competencies
Take a holistic history of the individual's health status Physically examine the individual to identify the health problem Order diagnostic tests Interpret the results of diagnostic tests Develop a differential diagnosis (a list of potential problems ranked by probability) and revise the list in accordance with the findings from the individual's history, physical examination, and diagnostics Use clinical reasoning to make decisions Prescribe medication to treat health problems Amend existing prescribed medication under the direction of a medical practitioner Prescribe non‐pharmacological interventions. Provide expert nursing care to individuals and their families Manage clinical risk, ensuring safe care Deliver prevention interventions to individuals, families, and wider communities Coordinate care for the individual with complex care needs Use e‐health and mobile health technologies	Direct clinical practice	Professional Values Clinical Decision making	Implement the fundamentals of nursing Apply context specific clinical competence Diagnose nursing care Diagnose advanced nursing care Make clinical decisions Implement scientific decision making Clinical decision‐making at an advanced level Deal with emergency care situations Implement nursing care Organise home care for patients Plan advanced nursing care Apply care in the long term Provide professional care in nursing Prescribe advanced nursing care Prescribe medication Evaluate nursing care Perform health assessment Initiate life preserving measures Ensure safety of healthcare users Respond to changing situations in healthcare Carry out nurse led discharge Use e‐health and mobile health technologies
15 Develop a person‐centred therapeutic relationship with individuals and their families 16 Support individuals and their families to make decisions about their care 17 Empower individuals to make decisions about their health	Counselling	Communication and Interpersonal	Apply person‐centred care Empower individuals, families and groups Interact with healthcare users. Listen actively Empathise with the healthcare user Develop a collaborative therapeutic relationship Promote inclusion Promote human rights
18 Utilise evidence to inform clinical practice 19 Identify potential research gaps from day‐to‐day practice 20 Contribute to research utilisation in everyday practice	Evidence‐based practice	Knowledge and educating others	Address the problem critically Conduct research into advanced nursing care Lead research activities in nursing Solve problems in healthcare Follow clinical guidelines
21 Use ethical reasoning to make decisions when faced with ethical dilemmas, e.g., a patient refuses care 22 Address ethical dilemmas by considering the best interest of individuals who are unable to make decisions for themselves 23 Support individuals and their right to self‐determination	Ethical decision‐making	Clinical decision making and communication and interpersonal	Advice on users' informed consent Screen patients for risk factors Advice on healthy lifestyles Educate on the prevention of illness Provide health education Develop advanced health promotion strategies Provide treatment strategies for challenges to human health
24 Identify modifiable risk factors 25 Empower individuals and families to change their behaviour using evidence 26 Educate individuals and families about their condition, its management and treatment 27 Coach individuals to take responsibility for self‐management 28 Educate individuals and families about prevention 29 Promote prevention in the community	Guidance and coaching		
30 Collaborate with other healthcare professionals to ensure continuity of care 31 Refer individuals to other health care professionals 32 Provide expert advice to other healthcare professionals	Collaboration	Team management and leadership	Provide nursing advice on healthcare Work in multi‐disciplinary health teams Deliver plans in relation to the transfer of care
33 Support the professional development of other staff members 34 Device programs of learning to support other staff members 35 Mentor other staff members in developing their competencies		Knowledge and Educating Others	Manage personal professional development Mentor other health professionals Participate in health personnel training
36 Lead clinical practice to improve care quality 37 Coordinate the team to ensure quality of care 38 Manage complex care 39 Manage distributed risk (risk that is not under your direct control) 40 Influence health policy based on your expertise of community health needs 41 Lead service changes by considering sustainable health care (e.g., care that addresses health needs without causing damage to environmental and other resources for future generations).	Leadership	Team management & Leadership	Adapt leadership styles in healthcare Co‐ordinate care Contribute to high level health strategic decisions Implement policy Inform policy makers of health related challenges Lead healthcare services changes Contribute to continuity in healthcare Comply with quality standards related to healthcare practice Analyse the quality of nursing care Comply with legislation related to healthcare Apply sustainability principles in health care Apply organisational techniques

### 5.2 Phase 2: Content Validity

A panel of ten experts was involved in evaluating the relevance and clarity of each item to assess the FCNs' advanced competencies. The experts included nurses working in an advanced practice nursing role within family practice and community. The initial set of items demonstrated a S‐CVI of 0.98 for relevance and 0.99 for clarity with no single item below 0.90. The set of items generated in phase 1 did not require any modification or deletion for clarity and relevance following the content validity assessment. The final version of the scale included 41 items, rated on a Likert scale from 1 (not at all) to 5 (very great extent).

### 5.3 Phase 3: Psychometric Testing

#### 5.3.1 Preliminary Analysis: Multivariate Normality

In this study, an initial number of 621 participants filled out the survey. Mardia's kurtosis was 2707.522, exceeding the critical value of 1763. The evaluation of the Mahalanobis distances identified 73 multivariate outliers, and, by deleting them, Mardia's kurtosis was 1490.018, hence verifying multivariate normality (Mikkonen, Tomietto, and Watson 2022). A t‐test comparison between multivariate outliers and normally distributed records highlighted statistically significant lower scores among multivariate outliers. This supports the decision to delete the outliers as they usually show a pattern compatible with flooring (as in this case) or ceiling effect in the distribution. By removing the 73 multivariate outliers, a final sample of 548 respondents was eligible for psychometric testing.

#### 5.3.2 Sample Description

The mean age of the participants was 50.1 years (SD = 8.5) with a minimum of 26 years and a maximum of 75 years. Overall, 90.7% (*n* = 497) of the respondents were female, and 50.5% (*n* = 277) worked in a GP practice, while the remaining were employed in community services. The majority were employed full‐time (60.9%, *n* = 334), and 81.8% (*n* = 448) declared spent at least 3 days per week in clinical practice with the patients. Looking at the year of registration as a nurse, 54.4% (*n* = 298) registered after 1998, and the remaining from 1973 to 1997. The completion of the Advanced Practice qualification occurred after 2019 for 54.6% of the sample.

#### 5.3.3 Scale Validation: Descriptives, EFA, and CFA

The initial scale, as administered after phase 2, showed both α and ω values of 0.94. The α and ω values did not show significant variation by deleting each item from the scale. Only item 2, “Physically examine the individual to identify the health problem(s)”, demonstrated an item‐to‐total correlation of 0.29. Most of the items had a mean score > 4.00, except items 40 and 41 (“Influence health policy based on your expertise of community health needs” and “Lead service changes by considering sustainable health care”) with respective a mean scores of 3.85 (SD = 1.07) and 3.91 (SD = 1.06). Skewness and kurtosis supported a normal distribution for most of the items with a tendency towards the highest scores in several items (Table 2).

**TABLE 2:** Descriptive statistics of the initial set of items (*n* = 548).

Items	Mean (±SD)	Min–Max	Skewness	Kurtosis	Item to total correlation	Cronbach's alpha if item deleted	McDonald's omega if item deleted
1. Take a holistic history of the individual's health status	4.91 ± 0.30	3–5	−3.46	12.00	0.46	0.94	0.94
2. Physically examine the individual to identify the health problem(s)	4.74 ± 0.61	1–5	−2.76	9.11	0.29	0.94	0.94
3. Order diagnostic tests	4.48 ± 0.77	1–5	−1.48	1.93	0.43	0.94	0.94
4. Interpret the results of diagnostic tests	4.43 ± 0.77	1–5	−1.24	1.04	0.44	0.94	0.94
5. Develop a differential diagnosis (a list of potential problems ranked by probability) and revise the list in accordance with the findings from the individual's history, physical examination, and diagnostics	4.76 ± 0.49	2–5	−2.06	4.03	0.43	0.94	0.94
6. Use clinical reasoning to make decisions	4.89 ± 0.33	3–5	−2.58	5.30	0.52	0.94	0.94
7. Prescribe medication to treat health problems	4.69 ± 0.65	1–5	−2.86	10.67	0.33	0.94	0.94
8. Amend existing prescribed medication under the direction of a medical practitioner	4.08 ± 1.17	1–5	−1.17	0.43	0.24	0.95	0.95
9. Prescribe non‐pharmacological interventions	4.50 ± 0.74	1–5	−1.45	1.88	0.46	0.94	0.94
10. Provide expert nursing care to individuals and their families	4.80 ± 0.47	2–5	−2.51	6.87	0.47	0.94	0.94
11. Manage clinical risk ensuring safe care	4.88 ± 0.34	3–5	−2.81	7.33	0.58	0.94	0.94
12. Deliver prevention activities and interventions to individuals, families, and wider communities	4.51 ± 0.76	1–5	−1.46	1.51	0.60	0.94	0.94
13. Coordinate care for the individuals with complex health and care needs	4.52 ± 0.71	1–5	−1.37	1.32	0.53	0.94	0.94
14. Where appropriate use e‐health and mobile health technologies	4.15 ± 0.92	1–5	−0.93	0.46	0.48	0.94	0.94
15. Develop a person‐centred therapeutic relationship with individuals and their families	4.83 ± 0.43	3–5	−2.47	5.57	0.56	0.94	0.94
16. Support individuals and their families to make decisions about their care	4.84 ± 0.39	3–5	−2.38	4.95	0.56	0.94	0.94
17. Empower individuals to make decisions about their health	4.86 ± 0.35	3–5	−2.36	4.44	0.61	0.94	0.94
18. Use evidence to inform clinical practice	4.91 ± 0.28	4–5	−2.93	6.58	0.50	0.94	0.94
19. Identify potential research and knowledge gaps from day‐to‐day practice	4.46 ± 0.71	2–5	−1.08	0.34	0.53	0.94	0.94
20. Contribute to research utilisation in everyday practice	4.02 ± 0.94	1–5	−0.67	−0.14	0.53	0.94	0.94
21. Use ethical reasoning to make decisions when faced with ethical dilemmas e.g., an individual refuses care	4.67 ± 0.54	3–5	−1.37	0.93	0.63	0.94	0.94
22. Address ethical dilemmas by considering the best interests of individuals who are unable to make decisions for themselves	4.60 ± 0.62	2–5	−1.38	1.22	0.59	0.94	0.94
23. Support individuals to exercise their right to self‐determination	4.70 ± 0.52	2–5	−1.55	1.96	0.60	0.94	0.94
24. Identify modifiable risk factors	4.66 ± 0.54	3–5	−1.31	0.75	0.66	0.94	0.94
25. Empower individuals and families to change their behaviour using evidence	4.61 ± 0.61	3–5	−1.30	0.60	0.67	0.94	0.94
26. Educate individuals and families about their condition, its management and treatment	4.80 ± 0.45	3–5	−2.07	3.56	0.62	0.94	0.94
27. Coach individuals to take greater responsibility for self‐management	4.55 ± 0.65	2–5	−1.17	0.37	0.61	0.94	0.94
28. Educate individuals and families about disease prevention	4.55 ± 0.67	2–5	−1.27	0.61	0.65	0.94	0.94
29. Promote prevention in the community	4.36 ± 0.83	2–5	−1.04	0.11	0.63	0.94	0.94
30. Collaborate with other healthcare professionals to ensure continuity of care	4.77 ± 0.46	3–5	−1.83	2.50	0.61	0.94	0.94
31. Refer individuals to other health professionals	4.70 ± 0.52	3–5	−1.54	1.47	0.50	0.94	0.94
32. Provide expert advice to other healthcare professionals	4.53 ± 0.68	1–5	−1.26	1.03	0.63	0.94	0.94
33. Support the professional development of other staff members	4.58 ± 0.66	1–5	−1.49	1.95	0.65	0.94	0.94
34. Devise programs of learning to support other staff members	4.11 ± 0.96	1–5	−0.93	0.38	0.64	0.94	0.94
35. Mentor other staff members in developing their competencies	4.42 ± 0.78	1–5	−1.19	0.73	0.59	0.94	0.94
36. Lead clinical practice to improve care quality	4.55 ± 0.69	1–5	−1.51	2.14	0.63	0.94	0.94
37. Coordinate the team to ensure quality care	4.26 ± 0.89	2–5	−1.05	0.51	0.57	0.94	0.94
38. Manage complex care	4.65 ± 0.61	1–5	−1.74	2.76	0.56	0.94	0.94
39. Manage distributed risk (risk that is not under your direct control)	4.06 ± 0.92	1–5	−0.66	−0.23	0.64	0.94	0.94
40. Influence health policy based on your expertise of community health needs	3.85 ± 1.07	1–5	−0.61	−0.37	0.64	0.94	0.94
41. Lead service changes by considering sustainable health care (e.g., care that addresses health needs without causing damage to environmental and other resources for future generations)	3.91 ± 1.06	1–5	−0.76	−0.06	0.62	0.94	0.94

When performing the EFA, both the KMO score (0.939) and the statistical significance of Bartlett's test of sphericity (χ^2^ = 13851.022, *p* < 0.001) confirmed the sample's adequacy. The initial solution identified 8 factors with an overall variance of 65.7%. However, significant cross‐loadings were detected for items 6, 30, 39, 32, 38, 12, 14, and 24. Moreover, items 9 and 8 showed loadings < 0.30. Accordingly, the deletion of these items was recommended. Furthermore, factors 6, 7 and 8 included only 1 or 2 items each, creating a psychometrically and theoretically unsustainable factorial solution. Accordingly, items 13, 31, 19, and 20 have also been considered for deletion (Table 3). By proceeding to delete these items and considering the items' theoretical consistency with the main construct, a 5‐factor solution comprising 27 items was identified and tested (Table 4). In the 5‐factor solution, factor 1 (7 items) aggregated the items linked to a “clinical care” domain, factor 2 (7 items) to a “leading practice” domain, factor 3 (5 items) to a “clinical reasoning” domain, factor 4 (5 items) to a “health promotion” domain, and factor 5 (3 items) to an “ethics” domain. Only item 26 in factor 4 showed a borderline cross‐loading. Each factor achieved good reliability (> 0.80) when considering both Cronbach's alpha and McDonald's omega. The overall scale showed a reliability score of 0.92 for both Cronbach's alpha and McDonald's omega. Item‐to‐total correlations, Cronbach's alpha, and McDonald's omega values if the item deleted confirmed the reliability of the final version of the scale (Table 5). This solution covered 66.3% of the overall variance, demonstrating that this factorial solution can explain more variation in the construct by being more parsimonious than the initial one.

**TABLE 3:** Exploratory factor analysis (Principal Axis Factoring, Rotation: Direct Oblimin) of the initial set of items (41 items, *n* = 548).

Items	Factor								Cross‐loading difference^a^
	1	2	3	4	5	6	7	8	
16. Support individuals and their families to make decisions about their care	**0.836**	−0.023	−0.039	−0.008	−0.036	0.057	0.004	0.033	0.78
15. Develop a person‐centred therapeutic relationship with individuals and their families	**0.746**	0.018	−0.021	−0.128	−0.046	0.174	−0.061	−0.019	0.57
17. Empower individuals to make decisions about their health	**0.728**	−0.033	−0.003	−0.092	0.019	−0.006	0.043	0.048	0.64
11. Manage clinical risk ensuring safe care	**0.687**	−0.007	0.142	0.051	0.119	−0.025	−0.049	0.034	0.54
18. Use evidence to inform clinical practice	**0.629**	0.024	0.056	−0.040	0.061	−0.198	0.085	0.116	0.43
1. Take a holistic history of the individual's health status	**0.479**	−0.054	0.114	0.056	0.052	0.009	0.014	0.060	0.36
6. Use clinical reasoning to make decisions	**0.455**	−0.099	**0.352**	0.062	0.143	−0.225	0.002	−0.013	**0.10**
10. Provide expert nursing care to individuals and their families	**0.426**	−0.026	−0.040	−0.086	0.054	0.155	0.109	0.018	0.27
30. Collaborate with other healthcare professionals to ensure continuity of care	**0.325**	−0.196	−0.055	−0.069	0.192	0.062	**0.313**	−0.002	**0.01**
35. Mentor other staff members in developing their competencies	0.012	**−0.791**	−0.048	−0.050	−0.016	−0.079	0.153	−0.024	0.64
34. Devise programs of learning to support other staff members	−0.064	**−0.779**	0.013	−0.056	0.004	−0.005	0.036	0.078	0.70
37. Co‐ordinate the team to ensure quality care	−0.023	**−0.769**	0.010	−0.031	−0.050	0.060	−0.008	0.001	0.71
36. Lead clinical practice to improve care quality	0.095	**−0.751**	0.024	−0.052	−0.005	−0.103	−0.078	0.023	0.65
33. Support the professional development of other staff members	0.084	**−0.717**	−0.007	−0.086	0.038	−0.134	0.194	−0.029	0.52
41. Lead service changes by considering sustainable health care (e.g., care that addresses health needs without causing damage to environmental and other resources for future generations)	−0.071	**−0.618**	0.033	−0.050	0.047	0.133	−0.240	0.230	0.38
40. Influence health policy based on your expertise of community health needs	−0.027	**−0.609**	0.034	0.007	0.050	0.211	−0.260	0.247	0.35
39. Manage distributed risk (risk that is not under your direct control)	0.025	**−0.508**	0.045	−0.061	0.168	0.252	−0.193	0.021	0.26
32. Provide expert advice to other healthcare professionals	0.082	**−0.504**	−0.024	−0.001	0.108	0.019	**0.305**	0.093	**0.20**
38. Manage complex care	0.276	**−0.313**	0.009	0.042	0.190	0.248	−0.013	−0.105	**0.04**
3. Order diagnostic tests	−0.083	0.014	**0.806**	−0.085	−0.042	0.044	0.012	0.102	0.70
4. Interpret the results of diagnostic tests	−0.046	−0.064	**0.733**	0.019	0.034	0.065	−0.005	0.064	0.67
2. Physically examine the individual to identify the health problem(s)	0.056	0.036	**0.686**	−0.043	−0.036	−0.074	−0.018	−0.034	0.61
5. Develop a differential diagnosis (a list of potential problems ranked by probability) and revise the list in accordance with the findings from the individual's history, physical examination and diagnostics	0.181	−0.002	**0.629**	0.005	0.105	−0.110	−0.150	0.005	0.45
7. Prescribe medication to treat health problems	0.026	0.027	**0.552**	−0.041	0.026	0.056	0.222	−0.107	0.33
28. Educate individuals and families about disease prevention	−0.002	−0.071	0.044	**−0.860**	−0.031	−0.013	0.017	0.036	0.79
27. Coach individuals to take greater responsibility for self‐management	−0.035	−0.066	0.037	**−0.781**	0.021	−0.029	0.026	0.033	0.71
29. Promote prevention in the community	−0.042	−0.102	−0.033	**−0.644**	0.047	0.117	0.011	0.134	0.51
25. Empower individuals and families to change their behaviour using evidence	0.060	−0.057	0.109	**−0.586**	0.241	−0.065	−0.011	−0.034	0.35
26. Educate individuals and families about their condition, its management, and treatment	0.262	−0.091	0.035	**−0.505**	0.144	−0.147	0.068	−0.079	0.24
12. Deliver prevention activities and interventions to individuals, families and wider communities	0.282	0.012	−0.058	**−0.388**	0.002	**0.376**	−0.031	0.094	**0.01**
14. Where appropriate, use e‐health and mobile health technologies	0.014	0.014	0.187	−0.254	0.029	0.174	−0.032	0.242	**0.01**
22. Address ethical dilemmas by considering the best interests of individuals who are unable to make decisions for themselves	−0.073	0.001	−0.044	0.046	**0.896**	0.071	0.065	0.039	0.82
21. Use ethical reasoning to make decisions when faced with ethical dilemmas, e.g., an individual refuses care	−0.029	0.040	0.027	−0.044	**0.790**	−0.039	0.029	0.149	0.64
23. Support individuals to exercise their right to self‐determination	0.104	−0.026	0.041	−0.171	**0.610**	−0.054	−0.113	−0.017	0.44
24. Identify modifiable risk factors	0.152	0.002	0.105	**−0.338**	**0.387**	0.037	−0.052	−0.012	**0.05**
13. Coordinate care for the individuals with complex health and care needs	0.201	−0.064	−0.048	−0.059	0.250	**0.539**	−0.002	−0.057	0.29
9. Prescribe non‐pharmacological interventions	0.088	−0.037	0.157	−0.117	0.067	0.262	0.151	0.058	**0.11**
31. Refer individuals to other health professionals	0.079	−0.076	0.118	−0.187	0.068	0.009	**0.456**	0.082	0.27
8. Amend existing prescribed medication under the direction of a medical practitioner	0.007	−0.064	0.193	0.081	0.015	0.184	0.217	0.041	**0.02**
19. Identify potential research and knowledge gaps from day‐to‐day practice	0.136	−0.005	−0.029	−0.059	0.047	−0.116	0.088	**0.723**	0.59
20. Contribute to research utilisation in everyday practice	0.014	−0.108	0.012	0.016	0.098	0.010	−0.029	**0.682**	0.57
Eigenvalues	14.321	3.567	2.222	1.858	1.426	1.332	1.158	1.067	
Variance (%)	34.929	8.701	5.420	4.533	3.478	3.249	2.825	2.603	
Cumulative variance (%)	34.929	43.630	49.050	53.583	57.060	60.309	63.134	65.737	

^a^ Cross‐loading difference: the difference in absolute values between the highest loading and the second highest loading (threshold < 0.20); Bold values represent factor loadings.

**TABLE 4:** Exploratory factor analysis for the 5‐factor solution (27 items, *n* = 548) (Principal Axis Factoring, Rotation: Direct Oblimin).

Items	Factor					Cross‐loading difference^a^
	1	2	3	4	5	
16. Support individuals and their families to make decisions about their care	**0.875**	−0.036	−0.047	0.020	0.017	0.83
17. Empower individuals to make decisions about their health	**0.766**	−0.041	−0.001	−0.071	−0.027	0.69
15. Develop a person‐centred therapeutic relationship with individuals and their families	**0.740**	0.004	−0.048	−0.111	0.005	0.63
11. Manage clinical risk ensuring safe care	**0.681**	−0.015	0.134	0.070	−0.123	0.55
18. Use evidence to inform clinical practice	**0.603**	0.032	0.092	−0.057	−0.051	0.51
1. Take a holistic history of the individual's health status	**0.476**	−0.083	0.117	0.065	−0.067	0.36
10. Provide expert nursing care to individuals and their families	**0.470**	−0.081	−0.026	−0.062	−0.063	0.39
34. Devise programs of learning to support other staff members	−0.017	**−0.846**	0.025	−0.007	0.004	0.82
37. Coordinate the team to ensure quality care	0.016	**−0.787**	0.004	0.003	0.060	0.73
35. Mentor other staff members in developing their competencies	0.101	**−0.778**	−0.035	−0.008	0.072	0.68
36. Lead clinical practice to improve care quality	0.111	**−0.729**	0.015	−0.029	0.032	0.62
40. Influence health policy based on your expertise of community health needs	−0.099	**−0.715**	0.014	−0.015	−0.172	0.54
41. Lead service changes by considering sustainable health care (e.g. care which addresses health needs without causing damage to environmental and other resources for future generations)	−0.139	**−0.713**	0.006	−0.066	−0.158	0.56
33. Support the professional development of other staff members	0.179	**−0.674**	0.024	−0.051	0.037	0.49
3. Order diagnostic tests	−0.114	−0.055	**0.862**	−0.050	0.015	0.75
4. Interpret the results of diagnostic tests	−0.068	−0.122	**0.785**	0.054	−0.052	0.66
2. Physically examine the individual to identify the health problem(s)	0.036	0.058	**0.704**	−0.016	0.063	0.64
5. Develop a differential diagnosis (a list of potential problems ranked by probability) and revise the list in accordance with the findings from the individual's history, physical examination, and diagnostics	0.113	0.035	**0.585**	−0.016	−0.102	0.47
7. Prescribe medication to treat health problems	0.094	0.050	**0.524**	−0.037	0.021	0.43
28. Educate individuals and families about disease prevention	−0.003	−0.016	0.025	**−0.921**	0.052	0.87
27. Coach individuals to take greater responsibility for self‐management	−0.030	−0.006	0.021	**−0.852**	0.013	0.82
29. Promote prevention in the community	−0.060	−0.142	−0.023	**−0.674**	−0.087	0.53
25. Empower individuals and families to change their behaviour using evidence	0.111	0.006	0.092	**−0.604**	−0.153	0.45
26. Educate individuals and families about their condition, its management and treatment	**0.321**	0.000	0.030	**−0.502**	−0.044	**0.18**
22. Address ethical dilemmas by considering the best interests of individuals who are unable to make decisions for themselves	0.038	−0.035	−0.028	0.035	**−0.861**	0.82
21. Use ethical reasoning to make decisions when faced with ethical dilemmas, e.g., individual refuses care	0.044	0.000	0.047	−0.069	**−0.778**	0.71
23. Support individuals to exercise their right to self‐determination	0.167	−0.006	0.024	−0.165	**−0.524**	0.36
Eigenvalues	9.974	3.144	1.999	1.575	1.214	
Variance (%)	36.942	11.646	7.406	5.833	4.497	
Cumulative variance (%)	36.942	48.588	55.994	61.827	66.324	

^a^ Cross‐loading difference: the difference in absolute values between the highest loading and the second highest loading (threshold < 0.20); Bold values represent factor loadings.

**TABLE 5:** Final scale (5 factors, 27 items): Descriptive statistics and internal consistency (Cronbach's alpha and McDonalds' omega). Overall *α* = 0.92; *ω* = 0.92. (*n* = 548).

Factors and items	Mean (±SD)	Min‐Max	Skewness	Kurtosis	Item to total correlation	Cronbach's alpha if item deleted	McDonald's omega if item deleted
**Factor 1: Clinical care (α = 0.87; ω = 0.88)**							
1. Take a holistic history of the individual's health status	4.91 ± 0.30	3–5	−3.46	12.00	0.53	0.87	0.88
10. Provide expert nursing care to individuals and their families	4.80 ± 0.47	2–5	−2.51	6.87	0.53	0.88	0.89
11. Manage clinical risk ensuring safe care	4.88 ± 0.34	3–5	−2.81	7.33	0.72	0.85	0.86
15. Develop a person‐centred therapeutic relationship with individuals and their families	4.83 ± 0.43	3–5	−2.47	5.57	0.72	0.85	0.85
16. Support individuals and their families to make decisions about their care	4.84 ± 0.39	3–5	−2.38	4.95	0.78	0.84	0.84
17. Empower individuals to make decisions about their health	4.86 ± 0.35	3–5	−2.36	4.44	0.77	0.84	0.85
18. Use evidence to inform clinical practice	4.91 ± 0.28	4–5	−2.93	6.58	0.62	0.86	0.87
**Factor 2: Leading practice (α = 0.91; ω = 0.91)**							
33. Support the professional development of other staff members	4.58 ± 0.66	1–5	−1.49	1.95	0.70	0.90	0.90
34. Devise programs of learning to support other staff members	4.11 ± 0.96	1–5	−0.93	0.38	0.79	0.89	0.89
35. Mentor other staff members in developing their competencies	4.42 ± 0.78	1–5	−1.19	0.73	0.73	0.89	0.90
36. Lead clinical practice to improve care quality	4.55 ± 0.69	1–5	−1.51	2.14	0.74	0.90	0.90
37. Coordinate the team to ensure quality care	4.26 ± 0.89	2–5	−1.05	0.51	0.72	0.89	0.90
40. Influence health policy based on your expertise of community health needs	3.85 ± 1.07	1–5	−0.61	−0.37	0.74	0.89	0.90
41. Lead service changes by considering sustainable health care (e.g., care that addresses health needs without causing damage to environmental and other resources for future generations)	3.91 ± 1.06	1–5	−0.76	−0.06	0.75	0.89	0.90
**Factor 3: Clinical reasoning (α = 0.83; ω = 0.84)**							
2. Physically examine the individual to identify the health problem(s)	4.74 ± 0.61	1–5	−2.76	9.11	0.62	0.79	0.82
3. Order diagnostic tests	4.48 ± 0.77	1–5	−1.48	1.93	0.74	0.75	0.76
4. Interpret the results of diagnostic tests	4.43 ± 0.77	1–5	−1.24	1.04	0.69	0.77	0.78
5. Develop a differential diagnosis (a list of potential problems ranked by probability) and revise the list in accordance with the findings from the individual's history, physical examination, and diagnostics	4.76 ± 0.49	2–5	−2.06	4.03	0.59	0.81	0.81
7. Prescribe medication to treat health problems	4.69 ± 0.65	1–5	−2.86	10.67	0.51	0.82	0.84
**Factor 4: Health promotion (α = 0.88; ω = 0.89)**							
25. Empower individuals and families to change their behaviour using evidence	4.61 ± 0.61	3–5	−1.30	0.60	0.71	0.86	0.88
26. Educate individuals and families about their condition, its management and treatment	4.80 ± 0.45	3–5	−2.07	3.56	0.65	0.88	0.89
27. Coach individuals to take greater responsibility for self‐management	4.55 ± 0.65	2–5	−1.17	0.37	0.79	0.84	0.85
28. Educate individuals and families about disease prevention	4.55 ± 0.67	2–5	−1.27	0.61	0.84	0.83	0.84
29. Promote prevention in the community	4.36 ± 0.83	2–5	−1.04	0.11	0.70	0.88	0.89
**Factor 5: Ethics (α = 0.85; ω = 0.86)**							
21. Use ethical reasoning to make decisions when faced with ethical dilemmas e.g., individual refuses care	4.67 ± 0.54	3–5	−1.37	0.93	0.76	0.75	^a^
22. Address ethical dilemmas by considering the best interests of individuals who are unable to make decisions for themselves	4.60 ± 0.62	2–5	−1.38	1.22	0.77	0.75	^a^
23. Support individuals to exercise their right to self‐determination	4.70 ± 0.52	2–5	−1.55	1.96	0.64	0.86	^a^

^a^ Cannot be estimated because the number of items is < 4.

Construct validity was then tested by performing a CFA on the 5‐factor solution. In detail, fit indices showed RMSEA = 0.076, SRMR = 0.058, CFI = 0.891 and TLI = 0.879, supporting the acceptable fit of this model. Another CFA model was tested by deleting item 26, as suggested by the EFA. The new model demonstrated improved fit indices, specifically RMSEA = 0.073, SRMR = 0.055, CFI = 0.903 and TLI = 0.890.

#### 5.3.4 Comparison Between GP Practice Nurses and Community Nurses

A secondary aim of this study was to identify any differences in the advanced competencies practiced by the advanced practice nurses working in a GP practice and a community setting. The sample was approximately equally distributed among the two settings, respectively *n* = 277 (50.5%) and *n* = 271 (49.5%). Items in factors 3 (clinical reasoning) and 4 (health promotion) consistently showed statistically significant higher scores among the general practice advanced practice nurses, except item 27. This is also represented by the overall mean scores of these two factors. In detail, factor 3 showed a mean score of 4.49 (SD = 0.60) among advanced practice nurses working in wider community roles and a mean of 4.75 (SD = 0.37) among GP advanced practice nurses (*p* < 0.001), and, in factor 4, a mean score of 4.51 (SD = 0.59) among APNs in wider community roles and a mean of 4.63 (SD = 0.47) among GP APNs (*p* = 0.012). Item 10 in factor 1 (clinical care) demonstrated a significantly higher score among community nurses, as well as items 33, 36, and 40 in factor 2 (leading practice) and item 22 in factor 5 (ethics). However, when considering the overall factors' scores, only factor 2 demonstrated a statistically significant higher score and, in detail, a mean score of 4.31 (SD = 0.69) among community nurses and a mean of 4.17 (SD = 0.73) among GP practice nurses (*p* = 0.021). Table 6 reports the detailed scores and p‐values for each of the 27 items of the 5‐factor scale.

**TABLE 6:** Difference between community nurses (*n* = 271) and GP practice nurses (*n* = 277) (df = 546).

Items	Group	Mean	SD	*t*‐test	*p*
**Factor 1: Clinical practice**	Community Nurses	4.88	0.28	1.22	0.223
	GP Practice Nurses	4.85	0.28		
1. Take a holistic history of the individual's health status	Community Nurses	4.94	0.27	2.04	0.042
	GP Practice Nurses	4.88	0.33		
10. Provide expert nursing care to individuals and their families	Community Nurses	4.85	0.41	2.422	**0.016**
	GP Practice Nurses	4.75	0.53		
11. Manage clinical risk ensuring safe care	Community Nurses	4.88	0.36	−0.341	0.733
	GP Practice Nurses	4.89	0.32		
15. Develop a person‐centred therapeutic relationship with individuals and their families	Community Nurses	4.84	0.43	0.793	0.428
	GP Practice Nurses	4.81	0.43		
16. Support individuals and their families to make decisions about their care	Community Nurses	4.87	0.36	1.334	0.183
	GP Practice Nurses	4.82	0.41		
17. Empower individuals to make decisions about their health	Community Nurses	4.86	0.37	−0.219	0.827
	GP Practice Nurses	4.87	0.34		
18. Use evidence to inform clinical practice	Community Nurses	4.91	0.28	−0.079	0.937
	GP Practice Nurses	4.91	0.28		
**Factor 2: Leading practice**	Community Nurses	4.31	0.69	2.306	**0.021**
	GP Practice Nurses	4.17	0.73		
33. Support the professional development of other staff members	Community Nurses	4.66	0.60	2.717	**0.007**
	GP Practice Nurses	4.51	0.70		
34. Devise programs of learning to support other staff members	Community Nurses	4.18	0.95	1.586	0.113
	GP Practice Nurses	4.05	0.97		
35. Mentor other staff members in developing their competencies	Community Nurses	4.47	0.77	1.558	0.120
	GP Practice Nurses	4.37	0.79		
36. Lead clinical practice to improve care quality	Community Nurses	4.62	0.66	2.634	**0.009**
	GP Practice Nurses	4.47	0.71		
37. Coordinate the team to ensure quality care	Community Nurses	4.32	0.86	1.56	0.119
	GP Practice Nurses	4.21	0.92		
40. Influence health policy based on your expertise of community health needs	Community Nurses	3.94	1.06	2.093	**0.037**
	GP Practice Nurses	3.75	1.06		
41. Lead service changes by considering sustainable health care	Community Nurses	3.98	1.10	1.425	0.155
	GP Practice Nurses	3.85	1.03		
**Factor 3: Clinical reasoning**	Community Nurses	4.49	0.60	−6.082	**< 0.001**
	GP Practice Nurses	4.75	0.37		
2. Physically examine the individual to identify the health problem(s)	Community Nurses	4.60	0.73	−5.189	**< 0.001**
	GP Practice Nurses	4.87	0.43		
3. Order diagnostic tests	Community Nurses	4.26	0.89	−6.861	**< 0.001**
	GP Practice Nurses	4.69	0.55		
4. Interpret the results of diagnostic tests	Community Nurses	4.29	0.88	−4.192	**< 0.001**
	GP Practice Nurses	4.56	0.61		
5. Develop a differential diagnosis	Community Nurses	4.68	0.57	−3.709	**< 0.001**
	GP Practice Nurses	4.84	0.39		
7. Prescribe medication to treat health problems	Community Nurses	4.61	0.79	−3.022	**0.003**
	GP Practice Nurses	4.77	0.47		
**Factor 4: Health promotion**	Community Nurses	4.51	0.59	−2.529	**0.012**
	GP Practice Nurses	4.63	0.47		
25. Empower individuals and families to change their behaviour using evidence	Community Nurses	4.55	0.66	−2.268	**0.024**
	GP Practice Nurses	4.66	0.55		
26. Educate individuals and families about their condition, its management, and treatment	Community Nurses	4.76	0.50	−1.65	**0.099**
	GP Practice Nurses	4.83	0.39		
27. Coach individuals to take greater responsibility for self‐management	Community Nurses	4.51	0.69	−1.229	0.220
	GP Practice Nurses	4.58	0.61		
28. Educate individuals and families about disease prevention	Community Nurses	4.45	0.76	−3.378	**0.001**
	GP Practice Nurses	4.65	0.56		
29. Promote prevention in the community	Community Nurses	4.29	0.89	−1.966	**0.05**
	GP Practice Nurses	4.43	0.75		
**Factor 5: Ethics**	Community Nurses	4.69	0.47	1.501	0.067
	GP Practice Nurses	4.62	0.51		
21. Use ethical reasoning to make decisions when faced with ethical dilemmas	Community Nurses	4.69	0.52	1.031	0.303
	GP Practice Nurses	4.65	0.56		
22. Address ethical dilemmas by considering the best interests of individuals	Community Nurses	4.68	0.56	2.821	**0.005**
	GP Practice Nurses	4.53	0.66		
23. Support individuals to exercise their right to self‐determination	Community Nurses	4.69	0.53	−0.149	0.881
	GP Practice Nurses	4.70	0.51		

*Note:* Bold values represent factor loadings.

## 6 Discussion

The analysis shows that the Family and Community Nursing Advanced Practice Scale has good internal consistency, with the final scale consisting of 5 factors. The first factor was “Clinical Care”, with a focus on patient assessment, care delivery, therapeutic communication, and empowering individuals alongside the management of risk. Factor 2 involved “Leading Practice” through leadership and team coordination, a focus on staff development and education, and leading policy influence and sustainability. Factor 3, “Clinical Reasoning”, involved examination and diagnostic reasoning alongside prescribing. Factor 4 was related to “Health Promotion” with a focus on prevention activities, health behaviour change, and patient education. The final factor had a focus on ethical reasoning, resolving dilemmas, and supporting individuals to make decisions.

This is the first study examining the scope of practice of United Kingdom‐based advanced nurse practitioners working in general practice and wider community settings. Some of the factors identified in this study are similar to those identified in other psychometric evaluations of advanced practice models, for example, in Jokiniemi et al. (2021), in which the Strong model has been tested. Jokiniemi et al. (2021) identified five factors with “direct care” broadly linking to “clinical practice” and “support for systems” broadly linking to the “leading practice”. The Strong model also includes an “education” factor and a “research” factor, both of which were absent from the assessment of the FCN Advanced Practice Scale, although the educational items formed part of the leadership of practice domain. The Strong model (Ackerman et al. 1996) was developed in the United States in the 1990s and is made up of 5 factors: direct care, education, research, support for systems, and publication and professional leadership. Although not explicitly referenced, the origins of the UK 4 pillars of advanced practice probably owe their origin to the Strong Model (Swarby, Reynolds, and Mortimore 2022). With the UK's leadership and management pillar being an amalgamation of support for systems and publication and professional leadership.

Factor 2 in this study related to leading practice and incorporated items related to educational support for team members, mentoring, and team leadership and coordination. The leadership role of advanced nurse practitioners has been extensively described (Heinen et al. 2019) and has been referred to as existing at four levels: system‐wide, organisational, team, and individual practice (Elliott et al. 2016). A scoping review by Elliott et al. (2016) identified that barriers often exist beyond the leadership of individual clinical practice that prevent advanced practice nurses from realising their full potential in this area. Such barriers include clinical pressures and a lack of mentorship and support from senior colleagues. Such pressures are exacerbated within general practice and primary care settings because of patient demand and more limited opportunities to influence at the system and organisational levels (British Thinks 2023). Given what we know about the limitations of such leadership roles, it is meaningful that in this study advanced practice nurses were able to lead wider teams, coordinate care, and seek to use their knowledge to influence policy.

A total of 14 items were deleted following initial analysis. Some of these can be explained as potential alternative forms of other items, e.g., amending a prescription under the supervision of a medical practitioner. Such activity would be rarely performed by an advanced practice nurse who was an independent prescriber. Other items form part of a wider range of activities; e.g., clinical reasoning is part of the process of patient history taking, examination, and creating a differential diagnosis list.

While items related to care coordination, the management of distributed risk, and managing complex care are probably more frequently performed by community nurses, some items scored with low levels of frequency were somewhat surprising given the study participants, these included the use of e‐health technologies to support patient management and the identification of modifiable risk factors.

One of the significant findings of this work relates to the fact that advanced practice nurses' involvement in a research pillar or domain was not confirmed in this study. In the UK, research is one of the four pillars of advanced practice, and it is also cited in other advanced practice models. Only one single research item remained in the final scale, that of using evidence to inform clinical practice. This is very similar to the requirement of a registered nurse not practicing at an advanced level. The other two research items were deleted as they did not load onto a specific factor. These were identifying potential research gaps from day‐to‐day practice and contributing to research utilisation in everyday practice. Advanced practice nurses' involvement in research is a known issue in the UK, and Fielding et al. (2022) described how practitioners often lack confidence when working in the research pillar. Fielding et al. (2022) went on to describe how advanced practice nurses have a role beyond the interpretation of research findings to inform practice and that they should be leading research implementation alongside conducting and leading research in the clinical setting. Dean (2023) argued that the pressures in the clinical practice pillar resulted in less time being available to develop engagement in the other areas of the advanced practice nurse role.

One potential solution to the issue of time for research in advanced practice roles is to explore the development of clinical academic careers for some practitioners through collaboration with other advanced practice nurses, this could have the potential to enhance research activity and engagement.

The FCN—APS suggests that engagement in each of the domains of practice is homogeneous between nurses working in general practice and those with roles in a wider community setting. This finding is interesting as the literature (Britain Thinks 2023) refers to the heterogeneous nature of advanced practice nurse roles. Nurses working in general practice often manage long‐term conditions and chronic diseases, and, as a result, the mean scores were significantly different for both clinical reasoning and health promotion than those for nurses working in the wider community. On the contrary, community nurses showed higher leadership competencies, possibly due to the clinical and organisational complexity they manage in their everyday practice, which requires the coordination of different stakeholders, services, and caregivers. There were no other statistically significant differences between the two groups for any of the other items in this study.

### 6.1 Strengths and Limitations

This is the first study to examine the role of advanced practice nurse in family and community practice. The work has led to the development and testing of a valid and reliable tool that can be used to assess other groups of community nurses. The study has identified a degree of homogeneity between advanced practice nurses working in general (family) practice and those working in other teams in the wider community.

The work is limited as it has only studied family and community nurses in the United Kingdom, where there is significant role specialisation and delineation. The results cannot be generalised to the wider family and community nurse roles across Europe. At the same time, the study does not provide insight into other groups of community nursing roles in the community and the degree of fit of those roles with advanced nursing practice. From the methodological point of view, the sample was adequate to test the scale's construct validity with both the EFA and the CFA. However, it would be recommended to perform the EFA and the CFA on different subsamples (e.g., by randomising in two groups the main sample). In this study, splitting the sample would have led to an inadequate item:participant ratio for the analyses. Future research should also explore the test–retest reliability of this scale in order for exploring its stability over time. The cross‐sectional design of this study did not allow to explore this aspect of the scale's reliability, and the online survey approach in data collection could have generated a self‐selection bias and a digital exclusion effect. Different data collection strategies are recommended in future studies to address these aspects.

### 6.2 Recommendations for Future Research and Practice

As several European countries have developed and implemented family and community nursing roles, there is scope to explore how these roles fit with the domains of advanced nursing practice. Given that prescribing rights and full practice authority, e.g., the ability to order diagnostic tests and refer to others, are not universal across all European countries, from a professional and organisational perspective, we would recommend adopting the 41‐item version of the scale in settings other than the UK or in contexts where these roles are regulated in a different way. This would allow mapping family and community nursing roles' competencies in different countries. From a research perspective, the 27‐item version of the scale demonstrated satisfactory reliability and validity, and there is a strong indication to adopt this version within the UK and other countries where family and community nurses' roles are regulated in the same way and exhibit the same range of competencies. Further research of this type would enable cross‐country comparison and would strengthen the evidence base about the scope of both family and community nurse practice and advanced practice more generally across European countries.

### 6.3 Implications for Policy and Practice

This study suggests that advanced nursing practice roles across family practice and wider community settings are homogeneous in terms of their scope of practice and activity. This supports health equity as it means that similar levels of care and service are available to individuals irrespective of the care setting.

The study highlights the need to strengthen the research domain of advanced practice nurses' practice. This can be achieved through research capacity building and the promotion of doctoral‐level study for advanced practice nurses. In addition, clinical academic careers would provide a useful route to enable advanced practice nurses to engage in research and potentially collaborate with other practitioners, thereby strengthening the research contribution of these roles.

Finally, the study has the potential to add to the body of knowledge to inform future advanced practice regulation in the United Kingdom by highlighting the scope of practice and showing that a wide range of roles across different practice settings meet the criteria for future UK advanced practice registration.

## 7 Conclusion

Advanced practice nurse roles have been implemented in the United Kingdom, often to address the specific needs of individual employers, raising concerns that the roles were often heterogeneous. At the same time, previous attempts to assess advanced practice nurse roles have concentrated on generic roles rather than roles in specific specialities such as family and community nursing practice. In addition, none of the previous studies had examined the frequency of involvement in different aspects of the role.

This study has attempted to address these issues by developing and psychometrically testing a new Family and Community Nursing—Advanced Practice Scale (FCN‐APS). The scale has good reliability, and analysis of the construct validity reveals five domains of advanced practice among this practitioner group. The domains are clinical care, leading practice, clinical reasoning, health promotion, and ethics. While the profile is broadly similar to the models in use across the four UK countries, it appears the research pillar is less developed and that this may be related to advanced practice nurse confidence and time to engage in research activities. Further analysis of the data allowed for the comparison between advanced practice nurses working in family (general) practice and those working in wider community roles. This analysis showed considerable homogeneity between the advanced practice nurses' work irrespective of the practice setting.

## Author Contributions

J.U., M.T., G.C., D.C., V.S., K.M., J.A., C.O.: conceptualisation. J.U., M.T., G.C., D.C., V.S., K.M., J.A.: methodology. J.U., M.T.: validation. J.U., C.O., M.T.: data curation. J.U., M.T., K.M., G.C., V.S., D.C.: writing – original. J.U., M.T., G.C., D.C., V.S., K.M., J.A., C.O.: writing – review and edit.

## Conflicts of Interest

The authors declare no conflicts of interest.

## Statistics Declarations

The authors have checked to make sure that our submission conforms, as applicable, to the journal's statistical guidelines. The authors affirm that the methods used in the data analyses are suitably applied to their data within their study design and context, and the statistical findings have been implemented and interpreted correctly. The author(s) agree to take responsibility for ensuring that the choice of statistical approach is appropriate and is conducted and interpreted correctly as a condition to submit to the journal.

## Supporting information


Appendix S1.

## Data Availability

The data that support the findings of this study are available from the corresponding author upon reasonable request.
